# Maintaining ocean ecosystem health with hydrocarbonoclastic microbes

**DOI:** 10.1093/ismeco/ycae135

**Published:** 2024-11-04

**Authors:** Wanpeng Wang, Bin Zhi, Yong Wang, Zongze Shao

**Affiliations:** State Key Laboratory Breeding Base of Marine Genetic Resources, Third Institute of Oceanography, Ministry of Natural Resources, Xiamen, Fujian 361005, China; Key Laboratory of Marine Genetic Resources, Third Institute of Oceanography, Ministry of Natural Resources, Xiamen, Fujian 361005, China; Key Laboratory of Marine Genetic Resources of Fujian Province, 184 Daxue Road, Xiamen, Fujian 361005, China; State Key Laboratory Breeding Base of Marine Genetic Resources, Third Institute of Oceanography, Ministry of Natural Resources, Xiamen, Fujian 361005, China; Key Laboratory of Marine Genetic Resources, Third Institute of Oceanography, Ministry of Natural Resources, Xiamen, Fujian 361005, China; Key Laboratory of Marine Genetic Resources of Fujian Province, 184 Daxue Road, Xiamen, Fujian 361005, China; State Key Laboratory Breeding Base of Marine Genetic Resources, Third Institute of Oceanography, Ministry of Natural Resources, Xiamen, Fujian 361005, China; Key Laboratory of Marine Genetic Resources, Third Institute of Oceanography, Ministry of Natural Resources, Xiamen, Fujian 361005, China; Key Laboratory of Marine Genetic Resources of Fujian Province, 184 Daxue Road, Xiamen, Fujian 361005, China; State Key Laboratory Breeding Base of Marine Genetic Resources, Third Institute of Oceanography, Ministry of Natural Resources, Xiamen, Fujian 361005, China; Key Laboratory of Marine Genetic Resources, Third Institute of Oceanography, Ministry of Natural Resources, Xiamen, Fujian 361005, China; Key Laboratory of Marine Genetic Resources of Fujian Province, 184 Daxue Road, Xiamen, Fujian 361005, China

**Keywords:** hydrocarbon pollution, hydrocarbon remediation, hydrocarbon-degrading microbes

## Abstract

Accidental spills and persisting hydrocarbon pollution caused by petroleum exploitation have deeply disrupted marine ecosystems, including those in the deep oceans and the Arctic Ocean. While physicochemical methods are available for emergency cleanup, microorganisms are ultimately responsible for mineralizing the hydrocarbons. The understanding of environmental effects on the composition and efficiency of hydrocarbon-degrading microbial communities has greatly improved current microorganism-based remediation strategies. This review summarizes recent findings on the physiology, metabolism, and ecology of marine obligate hydrocarbonoclastic microorganisms. Strategies for improved biotechnological solutions based on the use of hydrocarbon-degrading microbes are discussed for hydrocarbon remediation in marine water columns, sediments, beaches, and the Arctic.

## Introduction

**Figure 1:**
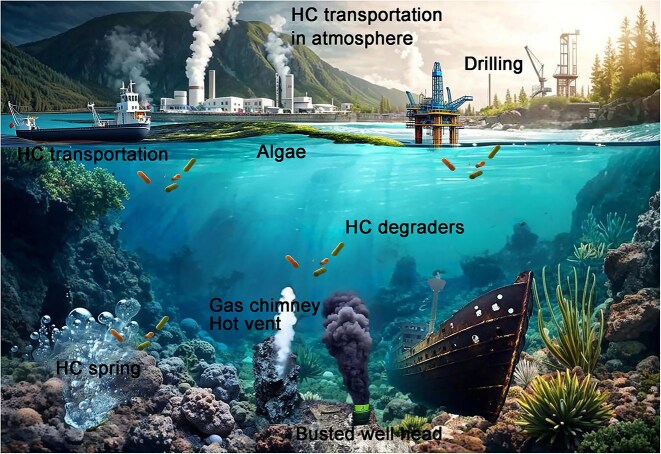
**Natural and anthropogenic sources and pathways of ocean hydrocarbons**. Natural and anthropogenic sources and pathways of ocean hydrocarbons are illustrated. HCs generated from algae, volcanos, gas vents, and human activity, including factory discharge, shipping leaks, and leaking oil wells will be slowly removed by HC-degrading microbes *in situ.*

Petroleum oil, a complex mixture of hydrocarbons (HCs) in gaseous, liquid, and solid forms, is the primary source of energy, chemicals, and material production, and therefore has a crucial role in the world economy. World consumption of petroleum has reached 30 billion barrels annually. Both natural and anthropogenic sources contributed to ocean HCs (Fig. 1). Massive shipping and offshore oil drilling poses a significant risk of accidents and oil spills. It is estimated that there are more than 120 million liters of oil spilled into marine environments every year from every step of the petroleum industry, including drilling, transportation, storage, manufacturing, and waste management, especially from leaky pipelines and sunken ships [1, 2]. Additionally, recent research showed that hydrothermal black smokers, which are cold hydrocarbon seeps from hydrocarbon springs, gas chimneys, mud volcanoes, and pockmarks at depths of 200–3500 m were estimated to produce more than 700 million liters of HCs per year [3, 4]. Furthermore, biological HC production should also be considered. For example, the global ocean production of chlorophyll A, the main pigment of photosynthesis that accounts for 0.3%–5% of the dry weight of microscopic algae and cyanobacteria, translates into about 308–771 million tons of HC annually [5]. Many microalgae produce HCs in substantial quantities in various forms [5–7], contributing to a high global de novo HC flux into the oceans, which amounts to 100–500-fold of that from fossil petroleum [8]. Anthropogenic oil spills, natural seeps, and biological production together would cover the world’s oceans with a 25–30-molecule-thick layer of HCs [8, 9]. In consequence, petroleum HCs are abundant in the ocean, and alkanes can even be found in waters that have been minimally contaminated by crude oil [8, 10].

The HC pollution of marine environments has been a global concern not only because of its disastrous direct effects on marine life but also because of its potential indirect damage to marine ecosystems, which will eventually threaten human health [11]. The hazardous effects of HC pollution include inflammation, immunosuppression, impaired phagocytosis, reproduction disorders, reef bleaching, carcinogenicity, teratogenicity, and mutagenicity [12, 13]. Therefore, technologies that could effectively remove HC pollutions at the shore or in the deep sea are highly sought after. In turn, this has led to a strong interest in natural biodegradation remediation based on oil-eating microorganisms for HC removal from the environment [1]. Specialized HC-degrading microbes, which are physiologically adapted and feed on crude oil, play an important role in protecting the ocean ecosystem from HC pollution [14]. Thus, studying these HC degraders could provide valuable knowledge on the mechanisms of natural HC contamination cleaning, and on strategies for developing new remediation methods for HC mineralization [15].

### Marine hydrocarbonoclastic microorganisms and biodegradation

The obligate hydrocarbonoclastic microorganisms (OHCM) are an important ecological protective group of marine microorganisms. OHCM almost exclusively utilize HCs as their source of energy and carbon, which is one of their metabolic peculiarities known as the “OHCM paradigm” [16, 17]. Bioremediation and the self-healing capacity of ecosystems against HC pollution depend heavily on marine OHCMs [17]. In order to mineralize HCs, electron receptors are generally required [18]. A variety of electron receptors can be employed by hydrocarbonoclastic bacteria to oxidize petroleum constituents, such as oxygen, nitrate, ferric iron, and sulfate [19]. The aerobic process, which involves oxygen-incorporating enzymes such as monooxygenases and dioxygenases, is generally more efficient and rapid than anaerobic process [20]. Under anaerobic conditions, HC degradation can be initiated by carbon-hydrogen bond activation via methylation, carboxylation, hydroxylation, and sub-terminal and terminal carbon addition [21]. However, the biological reactivity of HCs is dramatically reduced without oxygen [22]. Thus, anoxic marine sediments present a biochemical challenge for microbial degradation [20].

As evidenced during the last dramatic oil-spill disasters of the Deepwater Horizon (DWH) drilling platform, OHCMs serve a fundamental role in bioremediation of areas polluted by petroleum HCs [9]. For the estimates release of 4.1 million barrels oil, approximately half of the oil ascended to the ocean surface and was skimmed and flared, while the rest were aggregated in various forms, including marine oil snow, and subjected to biodegradation, which were considered the primary cleaning force in a four year follow up study [23]. To date, marine OHCMs exclusively belong to the *Gammaproteobacteria* class, which are subdivided into four orders: *Cellvibrionales* (*Porticoccus* [24]); *Nevskiales* (*Algiphilus* [25] and *Polycyclovorans* [26]); *Oceanospirillales* (*Alcanivorax* [27], *Neptunomonas* [28], *Oleibacter* [29], *Oleiphilus* [30], *Oleispira* [31], and *Thalassolituus* [32]), and *Thiothrichales* (*Cycloclasticus* [33]),and their taxonomic diversity has changed little at the higher taxa level.

OHCMs were originally classified as highly specialized hydrocarbonoclastic bacteria that degraded either aliphatic HCs (such as *Alcanivorax*, *Oleibacter*, *Oleiphilus*, *Oleispira*, and *Thalassolituus*)*,* or aromatic HCs (such as *Cycloclasticus* and *Neptunomonas* [16, 34]). It has been found that OHCMs are more metabolically versatile than previously believed. For example, in addition to the type species *Alcanivorax borkumensis* SK2^T^, the genus *Alcanivorax* currently includes 14 species with valid names (https://lpsn.dsmz.de/genus/alcanivorax). Some of them exhibit larger genome sizes than the strain SK2T, thereby utilizing a wider selection of growth substrates [17, 35, 36]. Specifically, different *Alcanivorax* strains utilize simple sugars (arabinose and glucose) [17] and aromatic compounds (benzene, toluene, chlorobenzene, and polycyclic aromatic hydrocarbon (PAH)) [17]. These findings expand the knowledge of the metabolic capability of OHCMs, historically known as narrow specialists in degradation of only aliphatic HCs (such as alkanes, cycloalkanes, and alkenes) [16, 27]. In concordance, the free-living *Cycloclasticus*, a well-established marine PAH-degrading genus that bloomed during the DWH oil spill, was found to be capable of degrading short-chain alkanes and establishing symbiosis with mussels and sponges isolated from asphalt-rich, deep-sea oil seeps [37–39]. These findings greatly expanded the role of keystone species in the degradation of marine HC pollution. Recent discoveries of novel OHCMs (*Algiphilus aromaticivorans*, *Polycyclovorans algicol*a, and *Porticoccus hydrocarbonoclasticus*) from eukaryotic phytoplankton also show diverse substrate patterns [24–26]. These novel OHCM isolates can utilize various HCs as their sole carbon sources for growth, including unbranched HCs (C_10_–C_16_), branched HCs (phytane and pristane), simple aromatic HCs (benzene, toluene, and p-xylene), and PAHs (naphthalene, fluorine, anthracene, phenanthrene, and pyrene) [17].

Oil degradation by archaea in marine systems is less clear. Although bacteria are believed to play a dominant role in oil degradation in oceans [40], archaea were reported in many studies to play a role as well [41–44]. Oil contamination in mangrove sediments differed from pristine sites based on denaturing gradient gel electrophoresis analysis of archaeal 16S rRNA genes, according to a field study [41]. Although the archaea community in the Gulf of Finland was not found to be interrupted by oil pollution in the coastal waters, they did exhibit significant changes in oil-contaminated sediments with higher concentration of *Halobacteriaceae* [42]. Enhanced archaeal cytochrome 450 and retinol metabolism activity in oil-contaminated sites indicated active oil degradation by archaea [42]. Furthermore, a recent survey of European Atlantic and Mediterranean coastal sediments also found the existence and abundance of the miscellaneous crenarchaeotic group to be correlated with oil-contaminated sediments [43]. In addition, HC degradation has been linked to methanogens in some marine systems. Methanogenesis was found to increase commensurately with HC degradation in phosphorus- or nitrogen-stimulated microcosms seeded with contaminated sediments [44]. In turn, the addition of methanol, acetate, or phthalic acid enhanced petroleum HC degradation in sediments [45, 46]. Overall, these results suggest that some archaea are involved in oil biodegradation in sediments.

Although marine OHCMs are often associated with petroleum-contaminated environments, where they are highly enriched, these organisms have also been found in unpolluted sites, such as various pristine areas and polar regions, which all seem to be untouched by HC pollution [9, 17]. Several biotic and abiotic sources of HCs can enter the ocean, albeit in small quantities, and sustain the emergence of OHCMs in the ocean, as noted in recent reviews [9, 17]. OHCM communities, both phycosphere-associated [17] and free-living, play a significant role in long-term and short-term HC cycles in the oceans, in which oil pollution and biogenic production of HCs by phytoplankton are contributors.

### Hydrocarbonoclastic microorganisms in hydrothermal environments

Microorganisms are the key cleaners for hydrocarbon pollutions in marine environments, including hydrothermal plumes and chimneys. For example, high abundance of *Alcanivorax* was found in hydrothermal plumes in the South Pacific [47]. Methane, alkane- and aromatics-oxidizing sulfate-reducing bacteria and archaea have also been found in these areas, indicating possible coupling between HCs degradation and sulfate reduction [48]. Resent laboratory study showed both enriched samples and mixtures of main isolated strains from hydrothermal plumes and chimneys could significantly degrades alkanes and PAHs in 60 days under lab setting [39]. Diversity detection showed *Alcanivorax, Glaciecola, Marinobacter, SUP05 clade, Cycloclasticus, Alteromonas, Sulfurimonas, Sulfurovum, Arcobacter, SAR324 clade, SAR202 clade,* and *Chloroflexi* in the plume samples, while *Alcanivorax, Marinobacter, Cycloclasticus,* and *Erythrobacter* proved to be the main degrader. On the other hand, *Sulfurovum, Sulfurimonas, Thiomicrospira, Nitrospira, Desulfurobacterium, Thermodesulfatator, Desulfobulbus, Pseudoalteromonas, Marinicella, Gallionella, Marinobacter, Halomonas, SAR202 clade,* and *Alcanivorax* were found in hydrothermal chimneys, while *Marinobacter, Sulfurimonas Halomonas Pseudoalteromonas, SAR202 clade,* and *SAR324 clade* proved to be the main degrader [39].

### Techniques to remove HC pollutions in marine ecosystems

#### Chemical and physical Cleanup

**Figure 2:**
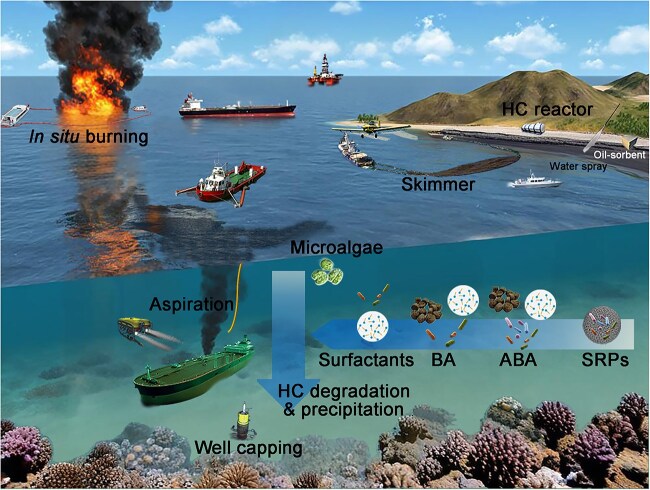
**Current technologies and biological remedies for hydrocarbon pollution in marine environments.** Hydrocarbon pollution on the ocean surface can be dispersed and collected with skimmers or undergo *in situ* burning, before undergoing volatilization or aggregation and deposition. Oil-sorbent materials are used to remove pollution on the beach with high-pressure water spray. Hydrocarbon pollution in the water column or bottom sediments can be aspirated into intake platforms while using wellhead capping to stop deep-sea oil well blowouts. In addition to natural HC-degrading microorganisms, bioaugmentation (BA) of lab-developed HC-degrading microbes can be used to enhance HC degradation rates along with associated nutrients and surfactants. Autochthonous bioaugmentation (ABA) using indigenous microorganisms enriched from contaminated habitats can greatly improve the adaptability of the microorganisms. Slow-release particles can be used to carry nutrients, biosurfactants, and HC-degrading microbes to enable continuous treatment at the polluted site. Microalgae can also contribute to the cleanup process by enhancing HC precipitation to the seafloor.

Currently, during an oil-spill emergency response, the oil is mechanically contained and recovered, chemical dispersants are applied, and shorelines are physically cleaned. An assessment of the extent of the spill would be taken first in a spill event to determine the best response. Water, shoreline, and sediment cleanup approaches are summarized here (Fig. 2).

Dispersants are generally applied in open waters and deep sea [49–51]. Dispersants, typically a surfactant, are often mixed with other solvents to better disperse spilled oil into small droplets assisted by natural ocean dynamics (Fig. 2) [51]. Thus, the oil slick does not reach the shoreline because oil droplets gradually sink into the water column instead of spreading along the ocean surface [51, 52]. In comparison with oil slicks, oil droplets provide more water–oil interface, resulting in a larger quantity of microorganisms being exposed to the oil surface [51, 53]. Dispersants can also prevent the light chain HCs from volatilizing and contaminating the atmosphere above [54]. Therefore, it is more effective to use dispersants immediately after a spill. Their effectiveness is also affected by temperature, salinity, and wave action [52]. However, dispersants themselves can cause secondary contamination because their chemical components are often highly toxic to marine life [51], including microorganisms [55]. The next technological goal in chemical remediation is to replace artificial surfactants with environmentally friendly biosurfactants, which are less toxic or even non-toxic to microbes [56].

The primary goal of physical attenuation is to contain and recover HCs with sorbents, barriers, skimmers, etc. [57] Usually, small spills, cleaning leftovers, or unreachable HC pollutants can be removed by natural or synthetic sorbents that capture oil either externally or internally [58]. Large-scale oil spills are generally treated with booms and skimmers [57]. The purpose of containment with barriers (fences, baffles, or booms) is typically to prevent oil from spreading over a larger surface area or impacting nearby marine resources, such as aquaculture surface waters. HCs are trapped as thick oil layers after containment by booms, facilitating subsequent skimming with skimmers [59]. A variety of skimmers, such as weir, oleophilic, and suction skimmers, have been developed to handle specific oil types, even when ice and debris are present [57].

In addition, special physical methods were tailored to match specific HC contamination (Fig. 2). For example, large-scale surface HC contamination could be burned *in situ* as an emergency action, with consideration of the toxicity assessments of burned residues. This strategy was adopted in several incidents with risky secondary pollution to human, including the DWH oil spill [60, 61]. HC contamination in coastlines without rich soil can be manually collected or flashed into the nearby water loaded with sorbent materials. However, containment that stops HCs from spreading is always the first consideration of a physical strategy, exemplified by the fact that all leaking treatment starts with capping the wellhead, including those of deep-sea spills (Fig. 2).

#### Bioremediation and natural attenuation

Although chemical and physical strategies can be applied immediately after oil spills, they have several drawbacks, such as the need for associated equipment and materials, incomplete elimination, and undesirable contamination from the cleaning chemicals [62]. Thus, bioremediation strategies are more appropriate for cleaning HC contamination.

The process of bioremediation uses the microorganisms that consume HCs as carbon and energy source [15]. Current bioremediation strategies including treating the HCs on site (*in situ*) or collecting the polluted materials and treating it in laboratory (*ex situ*) [63]. Therefore, it is necessary to understand microorganism viability, efficiency, and interacting mechanism for better bioremediation design. There are two types of marine microorganisms that degrade HCs: specialists and generalists, which are more capable of growing on low levels of HCs and higher levels of carbon sources, respectively [17, 34]. Compared to generalists, specialists are more active in HC cleaning, rather than exploiting and feeding on other nutrition, including bacteria, fungi, and archaea. Hydrocarbonoclastic bacteria were most studied for their ubiquity and prevalence. In return, the understanding of the mechanism of natural attenuation reveals many efficient strategies to accelerate the cleaning process. The main cultivated marine hydrocarbon-degrading microorganisms were summarized in Table 1.

**Table 1:** A comprehensive list of the main cultivated marine hydrocarbon-degrading microorganisms and their phylogenetic, physiological, and ecological features.

**A/AN**	**Genome**	**Microorganism**	**Phylogeny**	**Target substrate**	**Habitat/Ecology**	**Physiology**	**Refs**
A	Y	*Acinetobacter venetianus*	γ*-proteobacteria, Moraxellaceae*	*n*-alkanes	Surface water, sediment.	Biosurfactant producer	[64]
A	Y	*Alcanivorax borkumensis*	γ*-proteobacteria, Alcanivoracaceae*	*n*-alkanes	Seawater, sediment, beach sand, coastal salt marsh	Biosurfactant producer, OHCB	[27, 65–67]
A	Y	*Alcanivorax dieselolei*	γ*-proteobacteria, Alcanivoracaceae*	*n*-alkanes	Seawater, sediment	Resistance to mild pressure increase, OHCB	[68–70]
A	Y	*Alcanivorax pacificus*	γ*-proteobacteria, Alcanivoracaceae*	PAHs	Seawater, sediment		[71]
A	Y	*Alteromonas naphthalenivorans*	γ*-proteobacteria, Alteromonadaceae*	PAHs	Seawater, tidal flat sediment	*r*-strategist, fast growth in nitrogen-deficient seawater	[72]
A	Y	*Aurantiacibacter atlanticus*	α*-proteobacteria, Erythrobacteraceae*	PAHs	sediment	OHCB	[73]
A	Y	*Bacillus pumilus*	*Bacilli, Bacillaceae*	*n*-alkanes, PAHs	Sediment	Resistance to heavy metals	[74, 75]
A	N	*Bacillus stratosphericus*	*Bacilli, Bacillaceae*	PAHs, BTEX	Seawater	High metabolic versatility, biosurfactant producer	[76]
A	Y	*Celeribacter indicus*	α*-proteobacteria, Rhodobacteraceae*	PAHs	Sediment	OHCB	[77]
A	Y	*Cycloclasticus pugetii*	γ*-proteobacteria, Piscirickettsiaceae*	PAHs	Sediment	Highly efficient transport systems for the capture of nutrients and oligo-elements	[16, 33]
A	Y	*Cycloclasticus sp.* P1	γ*-proteobacteria, Piscirickettsiaceae*	PAHs	Sediment	moderately thermophilic, OHCB	[78]
A	Y	*Dietzia maris*	*Actinobacteria, Dietziaceae*	*n*-alkanes, PAHs	Seawater, deep sea hydrothermal field	Biosurfactant producer	[79, 80]
A	N	*Dothideomycetes- related taxa*	*Fungi*	PAHs	Beach sediment, tarballs, salt marshes	-	[81, 82]
A	N	*Halomonas halodurans; Halomonas organivorans*	γ*-proteobacteria, Halomonadaceae*	*n*-alkanes	Seawater, sediment	Key role in N metabolism to sustain degrading consortia	[83, 84]
A	Y	*Marinobacter hydrocarbonoclasticus*	γ*-proteobacteria, Alteromonadaceae*	*n*-alkanes, PAHs	Seawater, sediment	Biofilm producer; oil surface colonizers	[85–87]
A	Y	*Marinomonas profundimaris*	γ*-proteobacteria, Oceanospirillaceae*	PAHs	Arctic sediment	Cold-adapted	[88]
A	Y	*Nitratireductor pacificus* pht-3B	α*-proteobacteria, Phyllobacteriaceae*	PAHs	sediment	OHCB	[89]
A	N	*Oleibacter marinus*	γ*-proteobacteria, Oceanospirillaceae*	*n*-alkanes	Seawater	Adapted to tropical marine environments	[29, 90]
A	N	*Oleiphilus messinensis*	γ*-proteobacteria, Oleiphilaceae*	*n*-alkanes	Seawater, sediment	Biofilm producer on oil droplets, OHCB	[30]
A	Y	*Oleispira antarctica*	γ*-proteobacteria, Oceanospirillaceae*	*n*-alkanes	Seawater	Cold-adapted OHCB	[91]
A	N	*Rhodobacter sp. SS12.29; Rhodococcus sp. ice-oil-488 s*	α*-proteobacteria, Rhodobacteraceae*	PAHs	Seawater	Key role in reducing the accumulation of metabolites resulting from PAH degradation	[92]
A	Y	*Sphingobium sp.* C100	α*-proteobacteria, Sphingomonadaceae*	PAHs	Arctic sediment	Cold-adapted	[93]
A	N	*Sphingopixis sp.*	α*-proteobacteria, Sphingomonadaceae*	PAHs	Seawater	Key role in reducing the accumulation of metabolites resulting from PAH degradation	[92]
A	Y	*Thalassolituus oleivorans*	γ*-proteobacteria, Oceanospirillaceae*	*n*-alkanes	Surface seawaters, sediments, coastal and estuarine areas	OHCB	[94]
A/AN	Y	*Thalassospira profundimaris*	α*-proteobacteria, Thalassospiraceae*	PAHs	Seawater		[95]
A/AN	Y	*Pseudomonas pachastrellae*	γ*-proteobacteria, Pseudomonadaceae*	*n*-alkanes, PAHs	Sediment, beach sand	Bioemulsification activity	[65, 96, 97]
A/AN	Y	*Pseudomonas stutzeri*	γ*-proteobacteria, Pseudomonadaceae*	*n*-alkanes, PAHs, BTEX	Seawater, marsh and marine sediments, beach sand	Biofilm producer	[74, 97, 98]
AN	Y	*Archaeoglobus fulgidus*	*Euryarchaeota, Archaeoglobaceae*	*n*-alkanes	Shallow marine hydrothermal system	Extremophile	[99]
AN	Y	*Desulfatibacillum alkenivorans*	δ*-proteobacteria*, *Desulfobacteraceae*	*n*-alkanes	Sediment	High metabolic versatility for anaerobic alkane utilization	[100]
AN	N	*Desulfococcus oleovorans*	δ*-proteobacteria*, *Desulfobacteraceae*	*n*-alkanes, aromatic HCs	Sediment	Sulfate-reducing bacteria	[101, 102]
AN	N	*Desulfosarcina- Desulfococcus cluster strains*	δ*-proteobacteria Desulfobacteraceae*	Short chain *n*-alkanes	Sediments of marine HC seeps	Propane and butane degraders; sulfate-reducing bacteria	[103, 104]
AN	Y	*Ferroglobus placidus*	*Euryarchaeota, Archaeoglobaceae*	Aromatic HCs	Shallow marine hydrothermal system	Hyperthermophilic	[105, 106]
AN	Y	*Thermococcus sibiricus*	*Euryarchaeota, Thermococcaceae*	*n*-alkanes	Oil reservoir	High metabolic versatility	[107]

A: aerobic; AN: anaerobic; OHCB: obligate hydrocarbonoclastic bacteria, PAH: polycyclic aromatic hydrocarbons; Y/N: Y, genome available for at least one strain/ N, genome not available (genome availability checked on NCBI database on July 05, 2023).

Natural attenuation refers to the HC cleaning process by the indigenous microorganisms after oil spill. Many factors affect the efficiency of natural attenuation, including community structure and the interactions between microbes [1, 108]. Low HC solubility is one of the factors limiting HC degradation in seawater [1]. Microorganisms increase and modulate HC bioavailability according to environmental conditions by producing biosurfactants and modifying cell membrane hydrophobicity, such as *Alcanivorax* spp. [109]. The pathways by which HCs are broken down depend on oxygen availability. Aerobic HC degradation started with incorporating oxygen atoms with HC and generating alcohols, which could be turned into carboxylic acids and mineralized by β-oxidation [110]. In aerobic systems, alkane hydroxylases are the most studied enzymes, including *almA, alkB*, and *p450* [110–112]. The *alkB* gene, which became a biomarker for bioremediation, is often found in highly efficient hydrocarbon-degrading bacteria with multiple homologues [111]. Coexistence of multiple *alkB* genes in several alkane degraders contributes to a wider substrate range and better environmental adaptation [113, 114]. For PAHs, which are carcinogenic with poor biodegradability, ring-hydroxylating dioxygenases (RHDs) were identified as the oxygen incorporating enzyme [78]. The intermediate product will then go through dehydrogenation and a couple of ring cleavage and oxidation events to form catechol, which is further converted to biological precursor molecules such as pyruvate to enter the TCA cycle [78]. Under anaerobic conditions, alkylsuccinate synthases activate HC catabolism in sediments by adding fumarate to the secondary carbon [115].

There is still a great deal to learn about the metabolic mechanism of *in situ* HC bioremediation, particularly the environmental effects [116, 117]. Natural bioremediation usually starting with aliphatic HC degraders (e.g. *Alcanivorax* spp.) and following by the aggregation of aromatic HC degraders (e.g. *Cycloclasticus* spp.) [110]. Major factors affecting natural bioremediation including the community structure of the autochthonous microbes, temperature, pH, humidity and so on. Furthermore, bioremediation was usually applied after physical and chemical cleanup. Cleanup strategies for major historical oil spills were summarized in Table 2. The effectiveness of bioremediation in these events was hard to determined due to differences in strategies and environmental conditions. However, some cleaning strategies of these events were partially relied on natural attenuation.

**Table 2:** Cleanup strategies for major historical oil spills.

Time	Vessel	Location	Type of Oil	Estimated Oil Released	Cleanup Strategy	Note
1970	Arrow	Chedabucto Bay, Nova Scotia, Canada.	Bunker C (No. 6) fuel oil	14 700 tons	Physical methods were used; partially relied on natural attenuation [118, 119].	The first major oil spill in Canadian ocean waters, part of the oil in the sunken vessel began to leak in 2015.
1978	Amoco Cadiz	North Brittany shores. France	Arabian Light and Iranian Light crude oil	223 000 tons	Physical, chemical, and biological methods were used [120].	A massive oil spill with extensive contamination due to the weather.
1979	Ixtoc I	Bay of Campeche, Gulf of Mexico	Crude oil	475 000 tons	Physical and chemical methods were used [121].	The oil rig caught on fire, exploded, and leaked for 9 months.
1979	Atlantic Empress	NE of Trinidad and Tobago	Light crude oil	287 000 tons	Physical and chemical methods were used [122].	The tanker burned for 14 days.
1983	Castillo De Bellver	Atlantic, off Saldanha Bay, Cape Town, South Africa	Light crude oil	252 000 tons	Chemical dispersants were used [123].	The wind sent the oil offshore.
1983	Nowruz Oil Field	Arabian Gulf.	Heavy crude oil	272 000 tons	Physical methods were used [124].	A ship collided with a well platform due to the Iraq-Iran War.
1989	Exxon Valdez	Alaska Sea	North Slope Heavy Oil (API 29)	36 000 tons	Physical and biological methods were used. Bioremediation was used as a major strategy [125].	The second largest oil spill in US history.
1980–1989	Baffin Island Oil Spill Project	Cape Hatt, Canada	Lago Medio crude oil	30 m^3^	Chemical dispersants were used; partially relied on natural attenuation [126, 127].	Experiments to examine the physical and chemical fate of crude oil released into the Arctic Ocean and to determine the effectiveness of the use of dispersants.
1991	Haven	Ligurian Sea	Heavy Iranian crude oil	144 000 tons	Physical methods were used [128].	The tanker exploded and burned for 70 hours.
1991	Gulf War	Arabian Gulf.	Crude oil	120 000 000 tons	Physical, chemical, and biological methods were used [129, 130].	The largest oil spill in history.
2010	Deepwater Horizon	Gulf of Mexico	Light Louisiana Oil (API 35.2)	688 000 tons	Physical and chemical methods were used. Dispersants were applied [120].	The largest spill in US history.
2019	Unknown	Entire northeastern and part of the southeastern coastline of Brazil	Crude oil	More than 5000 tons	Physical methods were used; partially relied on natural attenuation [131, 132].	The most extensive spill in tropical oceans.

#### Bioremediation for sea water

Current ecosystem-friendly bioremediation technologies include biosurfactant amendments, biostimulation, and bioaugmentation. The addition of artificial and biological surfactants increases oil solubility in the polluted area and disperses HCs in the form of oil droplets, which allows HCs to be accessible to microorganisms without the production of biosurfactants (Fig. 2). Compared to artificial surfactants, biosurfactant is more ecofriendly due to its non-toxicity and biodegradability. Furthermore, biosurfactant is very stable under extreme temperature, pH, and salinity, compared to a dispersant [133]. To this day, biosurfactant is widely applied in pipeline transportation of oil, emulsified fuel production, recovery of reservoir oil, and HC biodegradation acceleration [133, 134]. *Acinetobacter*, *Alcanivorax*, *Bacillus*, and *Pseudomonas* are renowned suppliers of biosurfactant, with *Alcanivorax* predominating in HC polluted waters globally because of its ability to produce large quantities of glycolipid biosurfactant [53].

Among the most effective methods to enhance bacterial degradation of HC contamination is biostimulation, since it can encourage the growth of the degrading micro-community by adding nutrients and rebalancing the C/N/P ratio [135]. Biostimulation is mainly limited by nutrient delivery due to rapid leaching [136]. Key nutrients in the ocean, such as nitrogen, phosphate, and uric acid, are used to enhance the proliferation of HC degraders in the form of high solubility fertilizers [137]. The encapsulation of nutrients with slow-release particles, which packed with biosurfactants and HC degrading microbes, leads to greater biostimulation effectiveness (Fig. 2). Several bio-friendly polymers, such as cellulose, alginates, and hydrogel polymers, could be engineered into functional release capsules [137, 138]. Cryo-crosslinking alginate beads with an ethyl cellulose coating made by microinjection was recently shown to increase the load capacity of N/P fertilizers with a slow release capability [137]. In comparison to free cells, alginate-encapsulated diesel-degrading bacteria increased petroleum HC removal despite the addition of nutrients, and promoted heterotroph growth in addition to supporting HC degraders [137].

Bioaugmentation is often used to further enhance HC degradation by adding selected oil degraders cultured in the laboratory. A spilled HC mixture may be too complex for indigenous microbial populations to process, or local HC degraders may be insufficient. Thus, HC degradation can be enhanced by bioaugmentation with site-allochthonous microorganisms, although the long-term survival of these have been questioned [137]. Microorganisms from polluted habitats that showed good degrading efficiency are used for such autochthonous bioaugmentation (Fig. 2). A recent simulating study showed that bioaugmentation with augmented microorganisms further enhanced HC removal compared to a biostimulation-only strategy [139]. As an alternative to specific degraders, bioaugmentation can be implemented with HC-degrading genetic elements, which introduce remediation genes into indigenous microorganisms from an exogenous inoculant through horizontal transfer [140, 141]. Additionally, bioaugmentation with a microbial consortium has more advantages compared to a pure culture because of the advanced stability and reliability conferred by metabolic diversity. Studies showed that microbial consortia produced in laboratories with designed function strains could enhanced the degradation and tolerated wider environmental variation [142, 143].

HC pollution in the Arctic generally results from leaks or accidents during oil exploration, storage, transportation (shipping and pipeline transfer), surface runoff, and current long-range transportation. Marine ecosystems are affected significantly by oil-spill disasters, especially those that are extensive, such as the Alaska Exxon Valdez tanker oil spill in 1989, the Mexico Deepwater Horizon oil rig explosion in 2010, and the Russia Norilsk diesel fuel spill in 2020. Human error, illegal oil waste disposal, and shipping accidents caused these disasters [144].

It was discovered that the integrity of the Arctic ecosystem, including marine animals, fungus, and bacteria in the area, has been deeply harmed by natural gas and oil exploration [145]. There have been disruptions to growth, reproduction, immunity, and mobility among wildlife, marine animals, and birds [145]. The hazardous effects of HC pollution include inflammation, immunosuppression, impaired phagocytosis, reproduction disorders, reef bleaching, carcinogenicity, teratogenicity, and mutagenicity [12, 13], especially from aldehydes, monocyclic aromatic HCs, and PAHs, which are extremely carcinogenic [144]. Remediation of oil contamination in icy waters in the Arctic is extremely challenging and therefore oil spills have a wide range of negative impacts on the environment, especially in the case of PAHs, which pose the highest potential risk and toxicity of all carcinogenic HCs. Compared to PAHs, their derivative lighter HCs can be hydrophilic and soluble in water [144]. Insolubility, stability, and toxicity results in a lower degradation rate and make PAHs more likely to be bioconcentrated. The threat of exposure of toxic HCs on Arctic life has been increasing at an alarming rate, which makes it essential to routinely assess the biological effects, including the damage to wildlife, flora, and humans.

Temperature has a significant impact on biological activity. However, the degradation rates observed in warmer climates might be underestimated if extrapolated to the Arctic waters, as some local microbes could evolve to maintain activity in the permanently cold temperature [146]. There is no doubt that some HC degraders are well adapted to the icy temperatures in the Arctic and Antarctic seawater [17, 146, 147]. The psychrophilic bacterium *Oleispira Antarctica,* e.g. was isolated from the Antarctic seawater long before it was associated with alkane degradation [16, 91]. Other genera that have been isolated and identified as low temperature HC degraders include *Agreia*, *Arthrobacter*, *Halomonas*, *Marinobacter*, *Pseudoalteromonas*, *Pseudomonas*, *Psychrobacter*, *Rhodococcus*, and *Shewanella* [16, 17, 147].

Marine oil degradation at low temperatures has been intensively studied following the Arrow Spill (1970) and the Metula Spill (1980). However, recent research found other factors sometimes to be more important to biodegradation than low temperature. Leendert Vergeynst and colleagues identified a number of Arctic-specific environmental factors that affected HC degraders other than temperature, including water dynamics, sediment plumes, ice formation, and phytoplankton blooms [147].

### Biotechnological approaches to remediate oil-contaminated marine sediments

Sediments in the ocean are important sinks for HC pollutions. Great variety of processes contribute to the transport of petroleum from the water column to the seafloor, including chemical, physical, and microbiological processes. Both natural and artificial effects should be considered, such as weathering, particulate matter adsorption [148], and chemical dispersant additions. During DWH accident, 1.8–14% of the oil reached the sea floor according to a survey using biomarker, while a radiocarbon distribution method showed 0.5–9% [148]. Depending on site conditions, oil can transport through the upper sediment layers after sedimentation. The anoxic conditions of sediments limit oxidative biodegradation processes, which may lead to its persistence in sediments, in addition to the low bioavailability caused by strong sorption of HCs on hydrophobic sedimentary materials.

Traditionally, *in situ* bioremediation has been considered an effective and sustainable method for cleaning contaminated sediments. Several approaches have been used to enhance indigenous HC-degrading microbial communities in marine sediments [135]. Typically, these involve the addition of subsurface nutrients, electron receptors, and biosurfactants that limit degradation rates. Biosurfactants can be added to indigenous oil degraders to enhance their activity [134]. The addition of nitrogen and phosphorous encourages a flourishing natural biodegrading community in the sediment, making the HCs as the primary energy source and yielding rapid HC removal [135, 149]. As an oxygen-limited environment, the bioavailability of electron receptors such as O_2_, Fe^3+^, NO_3_^−^, and SO_4_^2−^, became a major factor affecting bioremediation efficiency. It has been proposed that different engineered approaches can be used to deliver oxygen to sediments to maintain aerobic conditions where petroleum HCs rapidly biodegrade. For example, oxygen injection, biosparging, bubbling, and mechanical agitation e diffusers was tested for *in situ* aeration for better bioremediation [150]. Despite its high effectiveness in enhancing the activity of degrading bacteria and removing HCs, most aeration system were proved to be labor/energy-intensive [150]. Alternatively, contaminated sediment can be treated by administering oxygen-generating chemicals such as CaO_2_ [151]. There are some limitations to this technique, however, including the rapid abiotic consumption of oxygen by reduced chemicals and the uncontrollable fast release of oxygen [151].

The development of an efficient bioelectrochemical system could also stimulate the biodegradation of HCs in sediments [152], making electricity generating degrader more appealing [153]. A classic bioelectrochemical system used a snorkel between the reductive sediment and the oxygen-rich water. In the anoxic sediment, the snorkel gathering electrons directly generated by microbial HC anaerobic oxidation and of inorganic chemical oxidation. Then the electrons are transferred to the oxygen-rich water, where they are acquired by oxygen and form water. In addition, electrons from the snorkel can anneal toxic sulfide generated by other bacteria, stimulating the growth of HC degraders [49]. According to this finding, the oil-spill snorkel may have a wide radius of influence beyond where the rod is positioned. This has major implications for practical applications. A laboratory-scale test of the oil-spill snorkel has demonstrated its feasibility. Since this technology has a low energy footprint, it could be applied to a wide variety of oil-spill situations for long-term HC remediation in remote areas.

## Conclusions and future perspectives

In nature, these ecological niches and their biogeochemical functions show pervasive and diverse potential for self-healing against HC pollution. Nonetheless, persistent HC biodegradation takes a long time, with very complex ecosystems involved, of which the primary driving factors are still unknown. In the future, it is crucial to better understand the basic mechanisms that govern the biodegradation of HCs in *in situ* conditions in order to design efficient strategies against HC contamination. As a result, we may be able to harness nature’s power to efficiently deal with oil spills near shores or out in the ocean and reduce the detrimental effects on both the environment and human society.

## Data Availability

Data sharing not applicable to this article as no datasets were generated or analyzed during the current study.
